# Complete Chloroplast Genome Sequence and Phylogenetic Analysis of the Medicinal Plant *Artemisia annua*

**DOI:** 10.3390/molecules22081330

**Published:** 2017-08-11

**Authors:** Xiaofeng Shen, Mingli Wu, Baosheng Liao, Zhixiang Liu, Rui Bai, Shuiming Xiao, Xiwen Li, Boli Zhang, Jiang Xu, Shilin Chen

**Affiliations:** 1 Institute of Chinese Materia Medica, Artemisinin Research Center, China Academy of Chinese Medical Sciences, Beijing 100700, China; 18384253631@163.com (X.S.); justuswu@163.com (M.W.); swjs082lbs@126.com (B.L.); zhixiangliu88@163.com (Z.L.); smxiao@icmm.ac.cn (S.X.); xwli@icmm.ac.cn (X.L.); zhangbolipr@163.com (B.Z.); 2 School of Chinese Materia Medica, Tianjin University of Traditional Chinese Medicine, Tianjin 300193, China; 3 College of Pharmacy, Hubei University of Chinese Medicine, Wuhan 430065, Hubei, China; 4 College of Pharmacy and Chemistry, Dali University, Dali 671000, Yunnan, China; 15010277446@163.com

**Keywords:** *Artemisia annua*, chloroplast genome, phylogeny

## Abstract

The complete chloroplast genome of *Artemisia annua* (Asteraceae), the primary source of artemisinin, was sequenced and analyzed. The *A. annua* cp genome is 150,995 bp, and harbors a pair of inverted repeat regions (IRa and IRb), of 24,850 bp each that separate large (LSC, 82,988 bp) and small (SSC, 18,267 bp) single-copy regions. Our annotation revealed that the *A. annua* cp genome contains 113 genes and 18 duplicated genes. The gene order in the SSC region of *A. annua* is inverted; this fact is consistent with the sequences of chloroplast genomes from three other *Artemisia* species. Fifteen (15) forward and seventeen (17) inverted repeats were detected in the genome. The existence of rich SSR loci in the genome suggests opportunities for future population genetics work on this anti-malarial medicinal plant. In *A. annua* cpDNA, the *rps19* gene was found in the LSC region rather than the IR region, and the *rps19* pseudogene was absent in the IR region. Sequence divergence analysis of five Asteraceae species indicated that the most highly divergent regions were found in the intergenic spacers, and that the differences between *A. annua* and *A. fukudo* were very slight. A phylogenetic analysis revealed a sister relationship between *A. annua* and *A. fukudo*. This study identified the unique characteristics of the *A. annua* cp genome. These results offer valuable information for future research on *Artemisia* species identification and for the selective breeding of *A. annua* with high pharmaceutical efficacy.

## 1. Introduction

*Artemisia annua*, an herbaceous annual with a strong volatile aroma, belongs to the genus Artemisia (Asteraceae). It is the sole natural source of the antimalarial drug artemisinin [1], and is cultivated as a high-value medicinal plant (Qing hao). Anti-malarial artemisinin combination therapy (ACT) has received strong interest from the global health community because of the efficacy of artemisinin and its derivatives [2]. Furthermore, the 2015 Nobel Prize for Physiology or Medicine was awarded to Professor Youyou Tu for the discovery of artemisinin [3]. However, there are concerns that the production of high-quality artemisinin may not be sufficient to meet future demand [2].

*A. annua* has a broad, global distribution and has many distinct locally-adapted ecotypes [4]. Beyond China, *A. annua* is also present in Eastern Europe, North America, and elsewhere in Asia [5]. However, the artemisinin content of *A. annua* ecotypes varies widely from region to region [5]. With the exception of a few rare high-artemisinin ecotypes found in China, the artemisinin content in *A. annua* ecotypes are generally insufficient (i.e., <1%) for commercialized extraction [6], and no other species been found to be suitable for mass production of artemisinin [1,7]. Oxygen released from chloroplasts in *A. annua* can upregulate the expression of genes involved in artemisinin biosynthesis, and can also catalyze artemisinin synthesis from dihydroartemisinin [8,9].

In addition to their role in photosynthesis, chloroplasts are also involved in cytoplasmic male sterility (CMS) [10] and secondary metabolic activities [11]. The chloroplast (cp) genome has a conserved quadripartite structure: a large single-copy (LSC) region, a small single-copy (SSC) region, and two inverted repeat (IR) regions. The majority of angiosperm cp genomes exhibit significant conservation of gene order and contents [12]. However, large-scale genome rearrangements and intron gains and losses have been identified in several angiosperm lineages [13,14,15]. A draft cp genome assembly for *A. annua* is of great importance for exploring putative links between *A. annua*’s chloroplast function and its adaptability and phytochemical characteristics.

The transcriptome sequences and genetic map of *A. annua* have been previously reported [16,17,18], but little is known about its cp genomic structure. Here we report the complete chloroplast genome sequence of *A. annua*, along with a characterization of long repeats and SSRs, and comparative analyses of the cp genome as a whole. Comparative analyses among cp genomes of other Asteraceae species revealed significant variation in genome size, highly divergent regions in intergenic spacers, as well as gene loss. Comprehensive cp genomic analyses will help to identify *Artemisia* species, provide insight into its evolutionary history, and improve the development of *A. annua* as a pharmacological resource [19,20].

## 2. Results and Discussion

### 2.1. Characteristics of A. annua cpDNA

**Figure 1:**
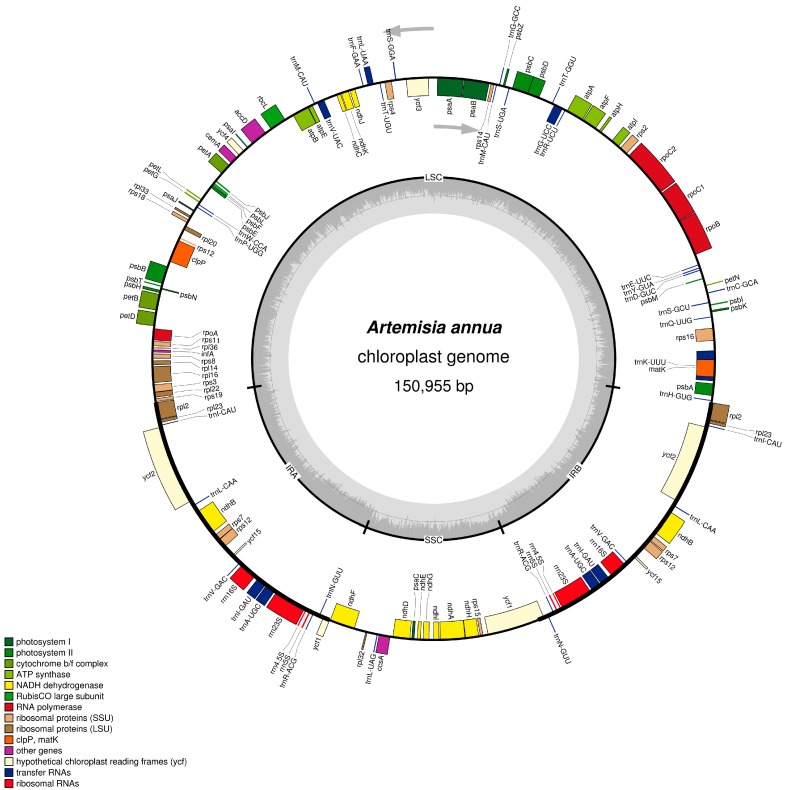
Gene map of the *A. annua* chloroplast genome. Genes drawn inside the circle are transcribed clockwise, and those outside are counterclockwise. Genes belonging to different functional groups are color-coded. The darker gray in the inner circle corresponds to GC content, while the lighter gray corresponds to AT content.

The complete cp genome of *A. annua* is 150,995 bp in size, with a pair of IR regions of 24,850 bp that separate a LSC region of 82,988 bp from a SSC region of 18,267 bp (Table 1 and Figure 1). The overall GC and AT content of the *A. annua* cp genome is 37.5% and 62.5%, respectively, which is similar to the cp genomes of other Asteraceae spp. [21,22,23]. The IR regions possess higher GC content (43%) than do the LSC (35.5%) or SSC regions (30.8%) (Table 1). Within the protein-coding regions (CDS), the AT content of the first, second, and third codon positions, is 54.6%, 62.4%, and 70.0%, respectively (Table 1). The bias toward a higher AT representation at the third codon position has been found to be common in other plant cp genomes [15,24], and this bias is used to discriminate cpDNA from nuclear and mitochondrial DNA [25]. The coding regions constitute 52.6% of the genome, and therefore the non-coding regions—including introns, pseudogenes, and intergenic spacers—account for 47.4%.

The *A. annua* cp genome encodes 113 predicted functional genes, including 80 protein-coding genes, 29 tRNA genes, and four rRNA genes (Table S1). In addition, there are 18 genes duplicated in the IR, making a total of 131 genes present in the *A. annua* cp genome (Figure 1). These genes have also been observed in *Artemisia frigida* [26]. Among these genes, seven protein-coding, seven tRNA, and all four rRNA genes are duplicated in the IR regions. The LSC region contains 62 protein-coding and 22 tRNA genes, whereas the SSC region contains one tRNA gene and 12 protein-coding genes.

Based on the sequences of protein-coding and tRNA genes, the frequency of codon usage was estimated for the *A. annua* cp genome and is summarized in Table 2. Together, all genes in the *A. annua* cp genome are encoded by 26,445 codons. Among these, leucine, with 2853 (10.7%) of the codons, is the most frequent amino acid in the cp genome, and cysteine, with 293 (1.1%), is the least frequent (Table 2). A- and U-ending codons were common. Except for *trnL-CAA*, all types of preferred synonymous codons (RSCU > 1) ended with A or U.

In total, there are 17 intron-containing genes, 15 (nine protein-coding and six tRNA genes) of which contain one intron, and two of which (*ycf3* and *clpP*) contain two introns (Table 3). The *trnK-UUU* has the largest intron (1860 bp), which itself contains the *matK* gene. The *rps12* gene is a trans-spliced gene with the 5′ end located in the LSC region and the duplicated 3′ ends in the IR regions. *Ycf3* is required for the stable accumulation of the photosystem I complex [27,28]. The intron gain in *ycf3* of *A. annua* may be useful for further studies of the mechanism of photosynthesis evolution, and of variation in singlet oxygen released by chloroplasts in from *Artemisia*.

Introns may contain “old code”—i.e., the part of a gene that loses its function during evolution. Several unicellular eukaryotes seem to experience selective pressures to lose introns. Therefore, the fact of intron gain and/or intron loss requires an evolutionary explanation. A common partial explanation for the range of intron densities is the random accumulation of introns in nuclear genomes over time after inheritance from an intron-poor ancestor. More experimental evidence is required to reveal whether the variation of the introns in the *A. annua* cp genome is related to adaptation to environmental stresses, or to facilitate artemisinin biosynthesis.

### 2.2. Long Repeat and SSR Analysis

For repeat structure analysis, 15 forward and 17 inverted repeats were detected in the *A. annua* cp genome (Table 4). Most of these repeats show lengths between 30 and 39 bp, while the *ycf2* gene possesses the two longest inverted repeats at 60 bp. Two repeats relevant to *psa* genes (No. 4 and 5) and three forward and three inverted repeats (No. 1–3, No. 16–18) in the intergenic spacers are distributed in the LSC region. Moreover, two forward and eight inverted repeats (No. 11 and 12, No. 22–29) associated with *ycf2*, two forward and two inverted repeats (No. 14 and 15, No. 31 and 32) in the intergenic spacers, are distributed in the IR region.

SSRs, well-known as microsatellites, are short (1–6 bp), tandemly repeated DNA sequences that are widely distributed throughout the genome. cpSSRs, uniparental in inheritance, have been widely employed in the analysis of plant population structure, diversity, differentiation and maternity analysis [29,30,31]. Here, the distribution of SSRs was analyzed for the *A. annua* cp genome, and 35 SSRs, most of them distributed in LSC, were identified. These included 31 mononucletide SSRs (88.57%), two dinucleotide SSRs (5.71%), and two trinucleotide SSR (5.71%) (Table 5). Sixteen of the 35 SSR loci were found in the intergenic regions, while the other 19 SSRs were located in genes. All 31 mononucleotide SSRs belonged to the A/T type. Our results are consistent with the hypothesis that cpSSRs are generally composed of short polyadenine (polyA) or polythymine (polyT) repeats and rarely contain tandem guanine (G) or cytosine (C) repeats. Thus, these SSRs contribute to the AT richness of cp genomes. cpSSRs have been important resources for the study of economically important plants and their relatives. Furthermore, the potential of cpSSRs to offer unique insights into species identification, genetic diversity, and evolutionary processes in wild plant species is quite tremendous [32]. Our results will provide cpSSR markers that can be used to examine genetic diversity in *A. annua* and its relative species, and to provide an efficient means by which to select germplasm with anti-malarial pharmaceutical efficacy.

### 2.3. Comparative Chloroplast Genomic Analysis

The whole cp genome sequence of *A. annua* was compared to those of *Artemisia fukudo*, *Lactuca sativa*, *Jacobaea vulgaris*, and *Cynara cornigera*. The cp genome size of *A. annua* is the second smallest among the five completed Asteraceae cp genomes. It is larger than *J. vulgaris* (150,689 bp) (Table S2), but smaller than the cp genomes of *A. fukudo*, *C. cornigera,* and *L. sativa* by 56 bp, 1595 bp, 1817 bp, respectively. *A. annua* has the smallest SSC region (18,267 bp) among these sequenced Asteraceae cp genomes. The next smallest SSC region is from *J. vulgaris*, with a size of 18,276 bp. There are no significant differences in sequence length between SSC or IR, and the variation in sequence length is the main reason that there is a difference in the length of the LSC region.

**Figure 2:**
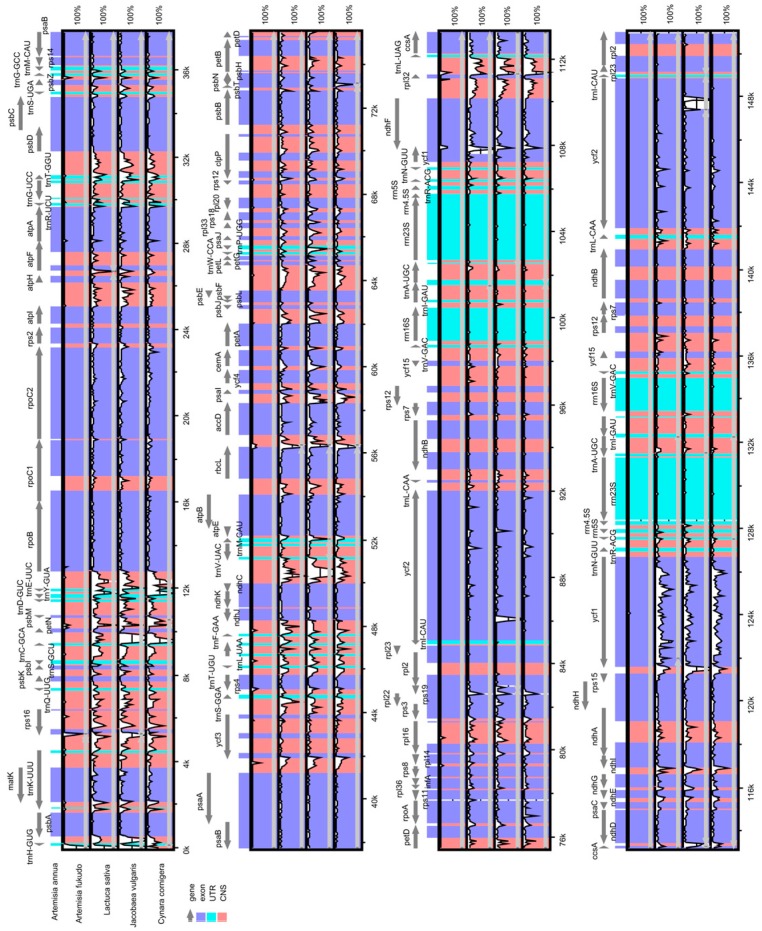
Comparison of five chloroplast genomes using mVISTA. Grey arrows and thick black lines above the alignment indicate gene orientation. Purple bars represent exons, blue bars represent UTRs, and pink bars represent non-coding sequences (CNS). The Y-scale axis represents the percent identity (shown: 50–100%). Genome regions are color-coded as either protein-coding exons, rRNAs, tRNAs, or conserved noncoding sequences (CNS).

Comparative genome analysis [33] permits the examination of how DNA sequences diverge among related species. The whole sequence identity of the five Asteraceae cp genomes was plotted using mVISTA, with the annotated *A. annua* cp genome as a reference (Figure 2). The comparison shows that the two IR regions are less divergent than the LSC and SSC regions. In addition, the coding regions are more conserved than the non-coding regions, and the highly divergent regions among the five cp genomes occur in the intergenic spacers, including *rnH-psbA*, *psbM-petN*, *trnC-GCA-petN*, *trnE-UUC-rpoB*, *trnY-GUA-trnE-UUC*, *trnV-UAC-ndhC*, *rbcL-accD*, *accD–psaI*, and *rpl32-trnL-UAG* in LSC, as well as *ndhI-ndhG* and *ycf1-rps15* in SSC. Similar results have been observed in other plant cp genomes [21,34]. Moreover, the most divergent coding regions are the *ndhF*, *ycf1*, and *ycf2* genes in five Asteraceae cp genomes. However, there is only a very slight difference between *A. annua* and *A. fukudo*. In our study, we observed that all eight rRNA genes are highly conserved.

### 2.4. IR Contraction and Expansion in the A. annua cp Genome

**Figure 3:**
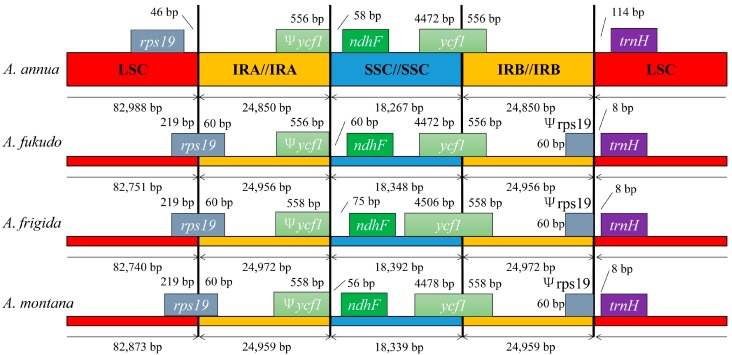
Comparison of the borders of the LSC, SSC, and IR regions among five chloroplast genomes. Ψ: pseudogenes, /: distance from the edge.

Although IRs are the most conserved regions of the cp genomes, contraction and expansion at the borders of IR regions are common evolutionary events, and are hypothesized to explain size differences between cp genomes [35,36]. Detailed comparisons of the IR-SSC and IR-LSC boundaries among four Asteraceae cp genomes (*Artemisia annua*, *Artemisia fukudo*, *Artemisi frigida*, and *Artemisia montana*) are presented in Figure 3. The IRb/SSC border is generally positioned between the *ycf1* pseudogene and the *ndhF* gene. The *ycf1* pseudogene has proven to be useful for analyzing cp genome variation in higher plants and algae [37]. The *ndhF* gene, related to photosynthesis, was found to be 56 bp, 58 bp, 60 bp, and 75 bp away from the IRb/SSC border, in *A. montana*, *A. annua*, *A. fukudo*, and *A. frigida*, respectively. However, some unique structural differences exist in the *A. annua* cp genome: the *trnH* gene is present at the longest distance (114 bp) from the LSC edge; the *rps19* pseudogene is absent in *A. annua* due to the contraction of the borders of the IR regions; the *rps19* gene was present in the LSC region due to the expansion of LSC. It has been reported that the *rps19* gene is one of the most abundant transcripts in the chloroplast’s genome [38]. The IR/LSC boundaries are not static among the cp genome in *Artemisia* species, but are dynamic processes confined to conservative expansions and contractions, which is similar to what has been found in other plants [39].

The comparison of cp genome size among examined Asteraceae species is displayed in Table S3. The length of the IR (24,850 bp) in *A. annua* is 106 bp smaller than that of *A. fukudo*, 122 bp smaller than that of *A. frigida*, and 109 bp smaller than that of *A. montana*. These differences may be related to the loss of *rps19* and *rps19* pseudogenes in *A. annua* IR regions. However, there are no significant differences in the length of the whole cp genome among the four Asteraceae cp genomes. The cp genome of *A. annua* (150,955 bp) is 56 bp smaller than that of *A. fukudo*, 121 bp smaller than that of *A. frigida*, and 175 bp smaller than that of *A. montana*. Non-functional DNA is rapidly deleted, resulting in the failure of pseudogenes to accumulate, which is the likely cause of this variation.

Pairwise cp genomic alignment between *A. annua* and the three *Artemisia* cp genomes (*A. frigida*, *A. fukudo*, and *A. montana*) revealed a high degree of synteny (Figures S1–S3). Previous work had reported that the cp genome of *A. frigida* had two inversion events in the LSC region, and at least one re-inversion event in the SSC [26]. Our results suggest that *A. annua* has similar sequence rearrangements. To further confirm the accuracy of the assembly and the gene order of the SSC in *A. annua*, four primers were designed to amplify the junctions of IRs and the LSC/SSC. These primers would create an amplicon by PCR amplification, which could then be analyzed via Sanger sequencing using the primers listed in Table S4. The inversion and re-inversion events in *A. annua* suggest that the SSC may be an active region for sequence rearrangements in plant cp genomes. Outside the Asteraceae [40,41], other angiosperms have been found to have an inverted SSC region, including *Piper cenocladum* [42], *Dioscorea elephantipes*, and *Chloranthus spicatus* [43]. Although chloroplast gene order is generally conserved in land plant genomes [44], many sequence rearrangements have been reported in cp genomes from a wide variety of different plant species, including inversions in the LSC region [45,46,47], IR contraction or expansions with inversions [48], and re-inversion in the SSC region. It has been proposed that sequence rearrangements in cp genomes are caused by intramolecular recombination events [49]. Sequence rearrangements that alter cp genome structure in related species may also provide genetic diversity information that can be used for molecular classification and evolution studies.

### 2.5. Phylogenetic Analysis

**Figure 4:**
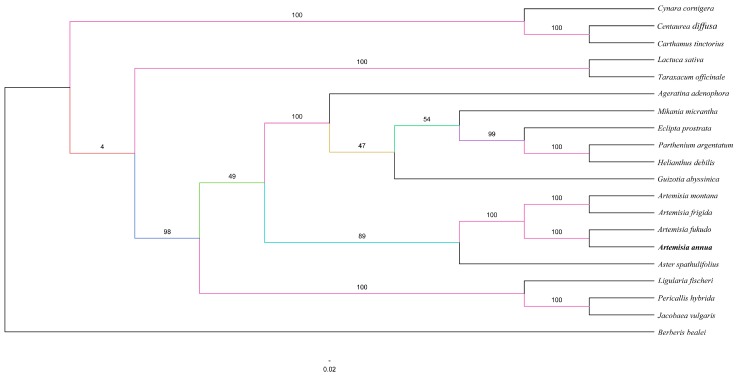
ML phylogenetic tree reconstruction 20 taxa of Asteraceae clade based on concatenated sequence from 50 chloroplast protein-coding genes. The position of *Artemisia annua* is indicated in block letter. *Berberis bealei* was set as the outgroup.

*A. annua* belongs to the tribe Anthemideae in the Asteraceae. Several studies have reported analyzes of the phylogenetic relationships within the Asteraceae based on chloroplast coding or non-coding sequences [50,51]. The availability of a completed *A. annua* cp genome provides us with sequence information that can be used to study the molecular evolution and phylogeny of *A. annua*. We performed multiple sequence alignments using 50 protein-coding genes commonly present in cp genome sequences in 20 Asteraceae species. One additional cp genome, *Berberis bealei* (Berberidaceae), was included as an outgroup (Figure 4). On the basis of a GTR + G + I nucleotide substitution model with 100% bootstrap values, as recommended by Jmodeltest, the ML phylogenetic results strongly supported the hypothesis that *A. annua* is the sister of the closely related species *Artemisia fukudo*. Furthermore, we hypothesized that *Artemisia fukudo* may have similar phytochemical properties [52].

## 3. Materials and Methods

### 3.1. DNA Sequencing, cp Genome Assembly, and Validation

Fresh *A. annua* leaves were collected from tissue cultured seedlings. Total DNA was extracted from approximately 10 g of fresh leaf tissue using the modified CTAB method [53]. The DNA concentration for each sample was estimated by measuring A260 using an ND-2000 spectrometer [54] (Nanodrop Technologies, Wilmington, DE, USA), and visual quality was assessed using agarose gel electrophoresis. Pure DNA was used to construct shotgun libraries (250 bp) according to the manufacturer’s instructions. Sequencing was performed by an Illumina Hiseq 1500 platform (San Diego, CA, USA). This resulted in approximately 100 Gb data. First, raw reads were trimmed by Fastqc. Next, we performed BLASTs between trimmed reads and reference sequences (*Artemisia frigida*) to extract cp-like reads [55]. Finally, the cp-like reads were used for sequence assembly with SOAPdenovo [56]. Sequence extension was executed using SSPACE [57], and gaps were filled using GapCloser [58]. To verify the assembly, the four junction regions between the IR regions and LSC/SSC were confirmed by PCR amplification and Sanger sequencing, using the primers listed in Table S4. The final cp genome of *A. annua* was submitted to GenBank (Accession Number: MF623173).

### 3.2. Gene Annotation and Sequence Analyses

The initial gene annotation was performed with CPGAVAS [59] (http://www.herbalgenomics.org/cpgavas) and further confirmation was performed using BLAST and DOGMA [60]. tRNA genes were identified by tRNAscanSE [61]. The circular cp genome map was drawn using the OGDRAWv1.2 [62] program (http://ogdraw.mpimp-golm.mpg.de/). To analyze the characteristics of variations in synonymous codon usage, relative synonymous codon usage values (RSCU), codon usage, and AT content were determined using MEGA5.2 [63].

### 3.3. Genome Comparison

MUMmer [64] was used to perform pairwise cp genomic alignment. The mVISTA [65] program in the Shuffle-LAGAN mode [66], was employed to compare the cp genome of *A. annua* with the cp genomes of *Artemisia fukudo*, *Lactuca sativa*, *Jacobaea vulgaris*, and *Cynara cornigera* (KU360270, AP007232, HQ234669 and KP842707), using the annotation of *A. annua* as the reference. MISA [67] was used to visualize the SSRs and REPuter [68] was used to visualize forward and inverted repeats.

### 3.4. Phylogenetic Analysis

A total of 19 complete cp genome sequences were downloaded from the NCBI Organelle Genome and Nucleotide Resources database. For the phylogenetic analysis, a set of 50 protein-coding genes shared in all 20 analyzed genomes was used. Genes were aligned by clustalw2 [69]. Jmodeltest 3.7 [70] was used to select the best model for ML (Maximum likelihood) analysis, and the phylogenetic tree was plotted using RAxML-HPC 2.7.6.3 on XSEDE at the CIPRES Science Gateway (http://www.phylo.org/). Bootstrap analysis was executed with 1000 replicates and TBR branch swapping. In addition, *Berberis bealei* was set as the outgroup.

## 4. Conclusions

Here we report the first complete cpDNA sequence of *A. annua*, an important medicinal plant. Compared to the cp genomes of three related Artemisia species, the cp genome of *A. annua* has the smallest size, while the genome structure and composition are similar. In addition, the cp genome of *A. annua* has an inverted SSC region, and is similar in that respect to most Asteraceae. However, a re-inversion event in the SSC region of the *A. annua* lineage suggests that the SSC might be an active region for inversion events in Asteraceae species. Repeated sequences, together with the aforementioned SSRs, are informative sources for the development of new molecular markers. Phylogenetic relationships among 20 Asteraceae species strongly supported the known taxonomic status of *A. annua* in Asteraceae and the sisterhood of the closely related species *A. fukudo*. The comprehensive data presented in this study provide insight into the evolutionary relationships between species of the genus Artemisia, and provide an assembly of a whole cp genome of *A. annua*, which may be useful for future breeding and further biological discoveries.

## Figures and Tables

**Table 1:** Base composition in the *A. annua* chloroplast genome.

Region		T (U) (%)	C (%)	A (%)	G (%)	Length (bp)
LSC		32.4	17.5	32.1	18.0	82,988
SSC		34.2	16.1	35.0	14.7	18,267
IRA		28.5	20.8	28.3	22.3	24,850
IRB		28.3	22.3	28.5	20.8	24,850
Total		31.3	18.7	31.2	18.8	150,955
CDS		31.6	17.6	30.7	20.1	79,335
	1st position	24.0	18.9	30.6	26.7	26,445
	2nd position	33.0	20.2	29.4	17.7	26,445
	3rd position	38.0	13.8	32.0	16.0	26,445

CDS: protein-coding regions.

**Table 2:** Codon-anticodon recognition patterns and codon usage of the *A. annua* chloroplast genome.

Amino Acid	Codon	No.	RSCU	tRNA	Amino Acid	Codon	No.	RSCU	tRNA
Phe	UUU	993	1.32		Tyr	UAU	811	1.64	
Phe	UUC	510	0.68	*trnF-GAA*	Tyr	UAC	178	0.36	*trnY-GUA*
Leu	UUA	890	1.87		Stop	UAA	52	1.77	
Leu	UUG	579	1.22	*trnL-CAA*	Stop	UAG	21	0.72	
Leu	CUU	622	1.31		His	CAU	471	1.51	
Leu	CUC	198	0.42		His	CAC	151	0.49	*trnH-GUG*
Leu	CUA	368	0.77		Gln	CAA	732	1.52	*trnQ-UUG*
Leu	CUG	196	0.41		Gln	CAG	230	0.48	
Ile	AUU	1092	1.47		Asn	AAU	1017	1.56	
Ile	AUC	433	0.58	*trnI-CAU*	Asn	AAC	287	0.44	
Ile	AUA	706	0.95		Lys	AAA	1042	1.47	
Met	AUG	633	1.00	*trnM-CAU*	Lys	AAG	371	0.53	
Val	GUU	512	1.44		Asp	GAU	868	1.61	
Val	GUC	174	0.49	*trnV-GAC*	Asp	GAC	213	0.39	*trnD-GUC*
Val	GUA	546	1.54		Glu	GAA	1001	1.50	*trnE-UUC*
Val	GUG	188	0.53		Glu	GAG	337	0.50	
Ser	UCU	588	1.74		Cys	UGU	202	1.38	
Ser	UCC	324	0.96	*trnS-GGA*	Cys	UGC	91	0.62	*trnC-GCA*
Ser	UCA	417	1.23	*trnS-UGA*	Stop	UGA	15	0.51	
Ser	UCG	167	0.49		Trp	UGG	462	1.00	*trnW-CCA*
Pro	CCU	441	1.58		Arg	CGU	350	1.33	*trnR-ACG*
Pro	CCC	188	0.67		Arg	CGC	107	0.41	
Pro	CCA	329	1.18	*trnP-UGG*	Arg	CGA	343	1.30	
Pro	CCG	159	0.57		Arg	CGG	124	0.47	
Thr	ACU	535	1.63		Arg	AGA	485	1.84	*trnR-UCU*
Thr	ACC	246	0.75	*trnT-GGU*	Arg	AGG	174	0.66	
Thr	ACA	411	1.25	*trnT-UGU*	Ser	AGU	410	1.21	
Thr	ACG	124	0.38		Ser	AGC	122	0.36	*trnS-GCU*
Ala	GCU	617	1.74		Gly	GGU	589	1.32	
Ala	GCC	228	0.64		Gly	GGC	189	0.42	*trnG-GCC*
Ala	GCA	415	1.17		Gly	GGA	707	1.58	
Ala	GCG	158	0.45		Gly	GGG	306	0.68	

RSCU: Relative Synonymous Codon Usage.

**Table 3:** The length of exons and introns in genes with introns in the *A. annua* chloroplast genome.

Gene	Location	Exon I (bp)	Intron I (bp)	Exon II (bp)	Intron II (bp)	Exon III (bp)
*trnK-UUU*	LSC	37	1860	35		
*trnG-UCC*	LSC	23	729	47		
*trnL-UAA*	LSC	37	424	50		
*trnV-UAC*	LSC	38	572	37		
*trnI-GAU*	IR	42	777	35		
*trnA-UGC*	IR	38	812	35		
rps12 *	LSC	232	535	26		114
*rps16*	LSC	40	876	185		
*rpl16*	LSC	9	1015	399		
*rpl2*	IR	394	626	470		
*rpoC1*	LSC	430	734	1640		
*ndhA*	SSC	556	1064	539		
*ndhB*	IR	777	670	756		
*ycf3*	SSC	127	700	230	735	153
*petB*	LSC	6	747	642		
*atpF*	LSC	145	699	410		
*clpP*	LSC	71	796	292	606	228

* The *rps12* gene is a trans-spliced gene with the 5′ end located in the LSC region and the duplicated 3′ ends in the IR regions.

**Table 4:** Long repeat sequences in the *A. annua* chloroplast genome.

ID	Repeat Start 1	Type	Size (bp)	Repeat Start 2	Mismatch (bp)	E-Value	Gene	Region
1	8544	F	32	34,909	−3	4.65E-05	IGS	LSC
2	28,063	F	31	29,661	−3	1.69E-04	IGS	LSC
3	28,070	F	30	29,666	−2	2.18E-05	IGS	LSC
4	38,054	F	32	40,278	−2	1.55E-06	*psaB*; *psaA*	LSC
5	38,065	F	30	40,289	−3	6.09E-04	*psaB*; *psaA*	LSC
6	43,070	F	41	96,883	−1	1.63E-13	*ycf3* (intron); IGS	LSC; IRA
7	43,072	F	39	118,107	−1	2.48E-12	*ycf3* (intron); *ndhA* (intron)	LSC; SSC
8	43,075	F	35	93,834	−3	9.59E-07	*ycf3* (intron); *ndhB* (intron)	LSC; IRA
9	66,346	F	30	98,046	−2	2.18E-05	IGS	LSC; IRA
11	86,539	F	30	147,378	−3	6.09E-04	*ycf2*	IRA; IRB
12	90,121	F	30	90,157	−1	5.00E-07	*ycf2*	IRA
13	96,885	F	39	118,107	0	2.12E-14	IGS; *ndhA* (intron)	IRA; SSC
14	105,777	F	30	105,809	−2	2.18E-05	IGS	IRA
15	128,104	F	30	128,136	−2	2.18E-05	IGS	IRB
16	8548	I	30	44,753	−2	2.18E-05	IGS	LSC
17	29,662	I	30	29,881	−2	2.18E-05	IGS	LSC
18	34,911	I	30	44,755	−1	5.00E-07	IGS	LSC
19	43,070	I	41	137,019	−1	1.63E-13	*ycf3* (intron); IGS	LSC; IRB
20	43,075	I	35	140,074	−3	9.59E-07	*ycf3* (intron); *ndhB* (intron)	LSC; IRB
21	66,346	I	30	135,867	−2	2.18E-05	IGS	LSC; IRB
22	90,109	I	60	143,756	−2	7.68E-23	*ycf2*	IRA; IRB
23	90,109	I	42	143,756	−2	2.57E-12	*ycf2*	IRA; IRB
24	90,121	I	30	143,756	−1	5.00E-07	*ycf2*	IRA; IRB
25	90,124	I	45	143,756	0	5.18E-18	*ycf2*	IRA; IRB
26	90,127	I	60	143,774	−2	7.68E-23	*ycf2*	IRA; IRB
27	90,142	I	45	143,774	0	5.18E-18	*ycf2*	IRA; IRB
28	90,145	I	42	143,792	−2	2.57E-12	*ycf2*	IRA; IRB
29	90,157	I	30	143,792	−1	5.00E-07	*ycf2*	IRA; IRB
30	105,777	I	30	128,104	−2	2.18E-05	IGS	IRA; IRB
31	105,809	I	30	128,136	−2	2.18E-05	IGS	IRA; IRB
32	118,107	I	39	137,019	0	2.12E-14	*ndhA* (intron); *rps12* (CDS)	SSC; IRB

F: Forward; I: Inverted; IGS: intergenic space; CDS: protein-coding regions.

**Table 5:** Simple sequence repeats in the *A. annua* chloroplast genome.

cpSSR ID	Repeat Motif	Length (bp)	Start	End	Region	Annotation
1	(A)15	15	3204	3218	LSC	*matK*
2	(A)14	14	3708	3721	LSC	
3	(A)10	10	6121	6130	LSC	
4	(T)10	10	9944	9953	LSC	
5	(A)10	10	13,630	13,639	LSC	*rpoB*
6	(A)12	12	20,826	20,837	LSC	*rpoC2*
7	(T)10	10	23,027	23,036	LSC	*rpoC2*
8	(A)11	11	26,289	26,299	LSC	*atpH*
9	(A)14	14	28,513	28,526	LSC	*atpA*
10	(A)11	11	39,312	39,322	LSC	*psaA*
11	(A)10	10	48,206	48,215	LSC	
12	(AT)6	12	52,028	52,039	LSC	
13	(T)14	14	53,085	53,098	LSC	*atpB*
14	(A)17	17	53,306	53,322	LSC	*atpB*
15	(A)19	19	54,902	54,920	LSC	*rbcL*
16	(A)10	10	56,832	56,841	LSC	
17	(A)14	14	57,920	57,933	LSC	*accD*
18	(A)11	11	59,654	59,664	LSC	*ycf4*
19	(T)10	10	59,775	59,784	LSC	*ycf4*
20	(T)10	10	64,476	64,485	LSC	
21	(T)10	10	64,902	64,911	LSC	
22	(A)11	11	66,255	66,265	LSC	
23	(T)10	10	69,525	69,534	LSC	
24	(A)14	14	70,210	70,223	LSC	
25	(T)10	10	71,655	71,664	LSC	*psbB*
26	(TA)6	12	72,640	72,651	LSC	*psbB*
27	(T)14	14	73,210	73,223	LSC	*psbN*
28	(A)15	15	80,929	80,943	LSC	
29	(T)10	10	81,209	81,218	LSC	
30	(T)11	11	101,234	101,244	IRA	
31	(GAA)5	15	108,039	108,053	SSC	*ndhF*
32	(TAA)5	15	117,240	117,254	SSC	*ndhI*
33	(T)10	10	118,903	118,912	SSC	
34	(A)14	14	121,936	121,949	SSC	*ycf1*
35	(A)11	11	132,700	132,710	IRB	

## Supplementary Materials

Table S1. Gene contents in the *Artemisia annua* chloroplast genome. (113 genes). Table S2. Size comparison of *Artemisia annua* chloroplast genomic regions and three other Asteraceae chloroplast genomes. Table S3. Size comparison of *Artemisia annua* chloroplast genomic regions and three other Artemisia chloroplast genomes. Table S4. Primers used for assembly validation. Figure S1. Chloroplast genomic alignment between *Artemisia annua* and *Artemisia frigida*. Figure S2. Chloroplast genomic alignment between *Artemisia annua* and *Artemisia fukudo*. Figure S3. Chloroplast genomic alignment between *Artemisia annua* and *Artemisia montana*.
