# Supplementary material for: Complete Chloroplast Genome Sequence and Phylogenetic Analysis of the Medicinal Plant Artemisia annua
Source: Molecules. 2017 Aug 11;22(8):1330. doi: 10.3390/molecules22081330 (PMC6152406; doi:10.3390/molecules22081330)
Supplement: Supplementary file 1 [file molecules-22-01330-s001.pdf]

Supplementary Materials

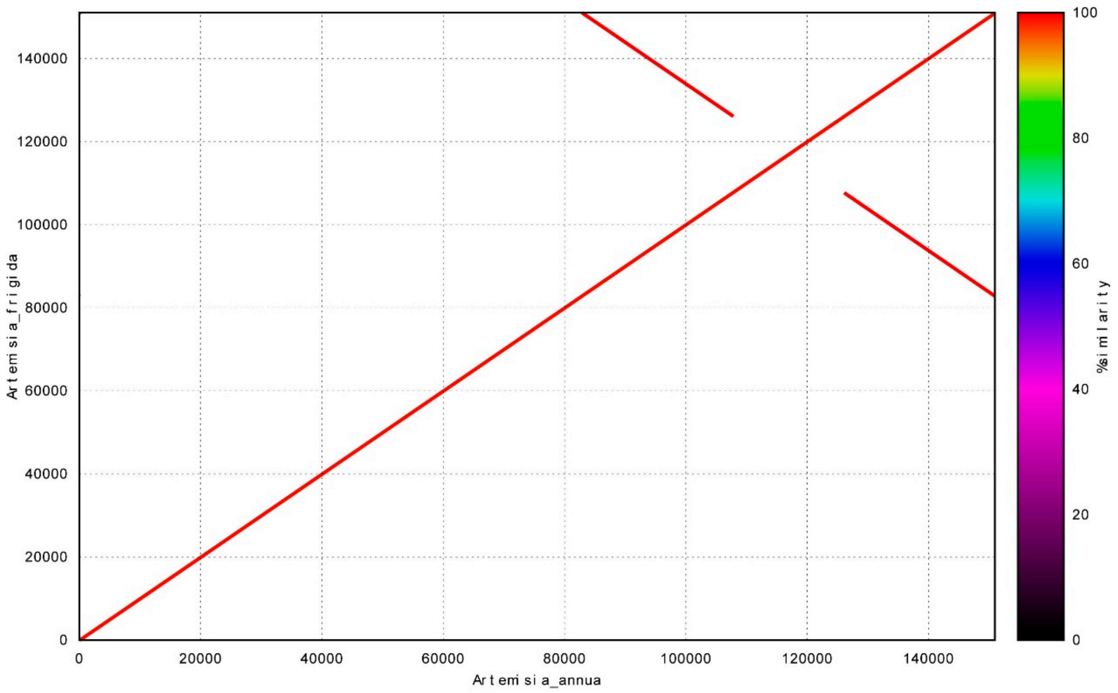

Figure S1. Chloroplast genomic alignment between *Artemisia annua* and *Artemisia frigida*.

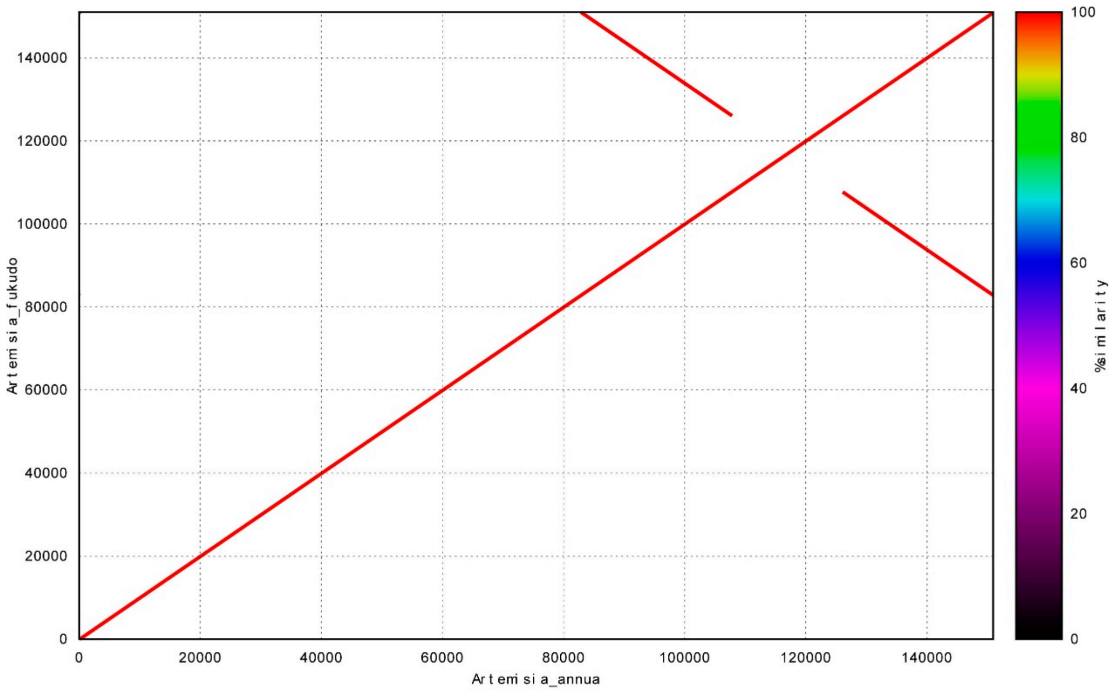

Figure S2. Chloroplast genomic alignment between *Artemisia annua* and *Artemisia fukudo*.

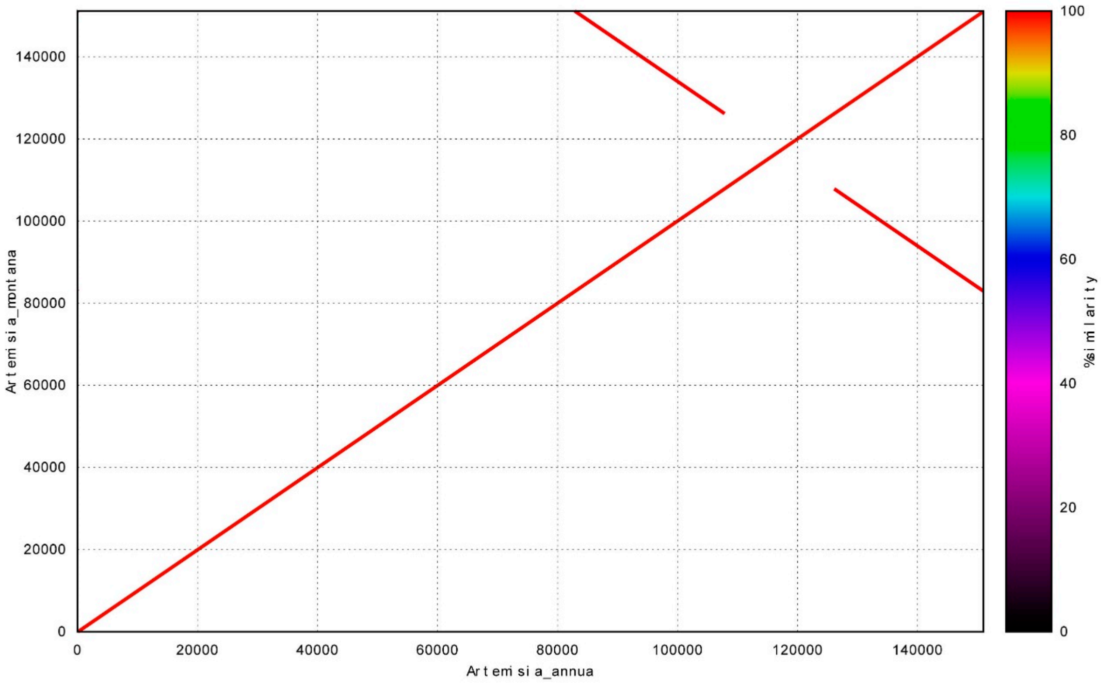

FigureS3. Chloroplast genomic alignment between *Artemisia annua* and *Artemisia montana*.

Table S1. Gene contents in *Artemisia annua* chloroplast genome (113 genes).

**Category for genes**	**Group of genes**	**Name of genes**
Self-replication	rRNA genes	*rrn16*[a], *rrn23*[a], *rrn5*[a], *rrn4.5*[a]
	tRNA genes	*trnH-GUG*, *trnK-UUU**, *trnQ-UUG*, *trnS-GCU*, *trnC-GCA*, *trnD-GUC*, *trnY-GUA*, *trnE-UUC*, *trnR-UCU*, *trnG-UCC**, *trnT-GGU*, *trnS-UGA*, *trnG-GCC*, *trnM-CAU*[a], *trnS-GGA*, *trnT-UGU*, *trnL-UAA**, *trnF-GAA*, *trnV-UAC**, *trnW-CCA*, *trnP-UGG*, *trnI-CAU*[a], *trnL-CAA*[a], *trnV-GAC*[a], *trnI-GAU**[,a], *trnA-UGC**[,a], *trnR-ACG*[a], *trnN-GUU*[a], *trnL-UAG*
	Small subunit of ribosome	*rps4*, *rps14*, *rps18*, *rps2*, *rps12***[,a], *rps11*, *rps8*, *rps3*, *rps19*, *rps7*[a], *rps15*, *rps16**
	Large subunit of ribosome	*rpl33*, *rpl20*, *rpl36*, *rpl14*, *rpl16**, *rpl22*, *rpl2**[,a], *rpl23*[a], *rpl32*
	DNA dependent RNA polymerase	*rpoB*, *rpoC1**, *rpoC2*, *rpoA*
	Translational initiation factor	*infA*
Genes for phytosynthesis	Subunits of NADH-dehydrogenase	*ndhA**, *ndhB**[,a], *ndhC*, *ndhD*, *ndhE*, *ndhF*, *ndhG*, *ndhH*, *ndhI*, *ndhJ*, *ndhK*
		psbA, *psaB*, *psaC*, *psaI*, *psaJ*, *ycf3***, *ycf4*
	Subunits od photosystem I	*psbA*, *psbB*, *psbC*, *psbD*, *psbE*, *psbF*, *psbH*, *psbI*, *psbJ*, *psbK*, *psbL*, *psbM*, *psbN*,
	Subunits od photosystem II	*psbT*, *psbZ*
		petN, *petA*, *petL*, *petG*, *petB**, *petD*
		atpI, *atpH*, *atpF**, *atpA*, *atpE*, *atpB*
	Subunits of cytochrome b/f complex	*rbcL*
	Subunits of ATP synthase	
	Large subunit of rubisco	
Other genes	Maturase	*matK*
	Protease	*clpP***
	Envelope membrane protein	*cemA*
	Subunit of Acetyl-CoA-carboxylase	*accD*
		ccsA
	C-type cytochrome synthesis gene	
Genes of unknown function	Open Reading Frames (ORF, ycf)	*ycf1*, *ycf15*[a], *ycf2*[a]
	Pseudo genes	*ycf1*

* Gene with one intron, ** Gene with two introns, [a] Gene with two copies.

Table S2. Size comparison of *Artemisia annua* chloroplast genomic regions with three other Asteraceae chloroplast genomes.

Species	Length (bp)			
	**Total genome**	**LSC**	**SSC**	**IR**
Artemisia annua	150,955	82,988	18,267	24,850
Artemisia fukudo	151,011	82,751	18,348	24,956
Lactuca sativa	152,772	84,105	18,599	25,034
Jacobaea vulgaris	150,689	82,855	18,276	24,779
Cynara cornigera	152,550	83,580	18,660	25,155

LSC, Large Single Copy; SSC, Small Single Copy; IR, Inverted Repeat.

Table S3. Size comparison of *Artemisia annua* chloroplast genomic regions with three other Artemisia chloroplast genomes.

Species	Length (bp)			
	**Total genome**	**LSC**	**SSC**	**IR**
Artemisia annua	150,955	82,988	18,267	24,850
Artemisia fukudo	151,011	82,751	18,348	24,956
Artemisia frigida	151,0n76	82,740	18,392	24,972
Artemisia montana	151,130	82,873	18,339	24,959

LSC, Large Single Copy; SSC, Small Single Copy; IR, Inverted Repeat.

Table S4. Primers used for assembly validation.

Primer	Sequence (5'>3')	Amplicon Size (bp)
LSC_IRa	TCAATTACTCTTCGCGCTTTGT	753
	CGGACAAGTGGGGAATGTTG	
IRa_SSC	CGATCTATTATGCGCCTCTGC	479
	GGGTGGGCGTATTTCCTCTT	
SSC_IRb	ATCTCGCTAACATTGAACTTGGT	401
	AATCAATTCGGTCGTTGTGGT	
IRb_LSC	CACTTGGAAAGGGTGGACAA	525
	CGTCGTTCGCCCAAATGAAA	
